# Coronavirus Disease 2019 Vaccine Impact on Rates of Severe Acute Respiratory Syndrome Coronavirus 2 Cases and Postvaccination Strain Sequences Among Health Care Workers at an Urban Academic Medical Center: A Prospective Cohort Study

**DOI:** 10.1093/ofid/ofab465

**Published:** 2021-09-17

**Authors:** Tara C Bouton, Sara Lodi, Jacquelyn Turcinovic, Beau Schaeffer, Sarah E Weber, Emily Quinn, Cathy Korn, Jacqueline Steiner, Elissa M Schechter-Perkins, Elizabeth Duffy, Elizabeth J Ragan, Bradford P Taylor, Nancy Miller, Ravin Davidoff, William P Hanage, John Connor, Cassandra Pierre, Karen R Jacobson

**Affiliations:** 1 Section of Infectious Diseases, Boston University School of Medicine and Boston Medical Center, Boston, Massachusetts, USA; 2 Department of Biostatistics, Boston University School of Public Health, Boston, Massachusetts, USA; 3 National Emerging Infectious Diseases Laboratories, Boston University, Boston, Massachusetts, USA; 4 Department of Epidemiology, Harvard T. H. Chan School of Public Health, Boston, Massachusetts, USA; 5 Biostatistics and Epidemiology Data Analytics Center, Boston University School of Public Health, Boston, Massachusetts, USA; 6 Department of Infection Control, Boston Medical Center, Boston, Massachusetts, USA; 7 Department of Emergency Medicine, Boston University School of Medicine and Boston Medical Center, Boston, Massachusetts, USA; 8 Department of Pathology and Laboratory Medicine, Boston University School of Medicine and Boston Medical Center, Boston, Massachusetts, USA; 9 Boston Medical Center and Boston University School of Medicine, Boston, Massachusetts, USA; 10 Department of Microbiology, Boston University School of Medicine, Boston, Massachusetts, USA

**Keywords:** COVID-19 vaccine, infection control, SARS-CoV-2 infections, viral evolution, whole genome sequencing

## Abstract

**Background:**

Coronavirus disease 2019 (COVID-19) vaccine trials and post-implementation data suggest that vaccination decreases infections. We examine vaccination’s impact on severe acute respiratory syndrome coronavirus 2 (SARS-CoV-2) case rates and viral diversity among health care workers (HCWs) during a high community prevalence period.

**Methods:**

In this prospective cohort study, HCW received 2 doses of BNT162b2 or mRNA-1273. We included confirmed cases among HCWs from 9 December 2020 to 23 February 2021. Weekly SARS-CoV-2 rates per 100,000 person-days and by time from first injection (1–14 and ≥15 days) were compared with surrounding community rates. Viral genomes were sequenced.

**Results:**

SARS-CoV-2 cases occurred in 1.4% (96/7109) of HCWs given at least a first dose and 0.3% (17/5913) of HCWs given both vaccine doses. Adjusted rate ratios (95% confidence intervals) were 0.73 (.53–1.00) 1–14 days and 0.18 (.10–.32) ≥15 days from first dose. HCW ≥15 days from initial dose compared to 1-14 days were more often older (46 vs 38 years, *P* = .007), Latinx (10% vs 8%, *P* = .03), and asymptomatic (48% vs 11%, *P* = .0002). SARS-CoV-2 rates among HCWs fell below the surrounding community, an 18% vs 11% weekly decrease, respectively (*P* = .14). Comparison of 50 genomes from post–first dose cases did not indicate selection pressure toward known spike antibody escape mutations.

**Conclusions:**

Our results indicate an early positive impact of vaccines on SARS-CoV-2 case rates. Post-vaccination isolates did not show unusual genetic diversity or selection for mutations of concern.

## Teaser

Cases of SARS-CoV-2 among health care workers dropped rapidly with COVID-19 vaccination. Sequencing 50 breakthrough infections (overwhelmingly in 14 days after first dose) showed no clear sign of any differences in spike protein compared with time-matched, unvaccinated control sequences.

On 15 December 2020, Boston Medical Center (BMC), an urban, safety-net hospital, started offering its 10 590 health care workers (HCWs) the severe acute respiratory syndrome coronavirus 2 (SARS-CoV-2) vaccines BNT162b2 (Pfizer/BioNTech) and then mRNA-1273 (Moderna). The vaccination campaign coincided with Massachusetts’ second surge, which peaked at 6000 new daily coronavirus disease 2019 (COVID-19) cases [1]. Vaccine was not available to anyone in the general population until 1 February 2021. This allowed examination of vaccine effectiveness during a period of higher prevalence and increased SARS-CoV-2 viral diversity than in the initial clinical trials [2, 3]. Concerns exist that newer SARS-CoV-2 variants have increased infectivity and modest decrease in neutralizing activity, and may impact vaccine effectiveness via escape from vaccine-induced immunity, specifically by mutations in the spike protein [4]. Assessment of viral genome sequencing of time-matched cases from vaccinated and unvaccinated individuals is needed to see whether such selection is evident.

Our aims were 3-fold: (1) to compare infection rates among HCWs who did and did not receive a SARS-CoV-2 vaccine; (2) to compare infection rates over time between HCWs and the surrounding community in the months following the BMC vaccination initiative; and (3) to compare genomic and spike protein mutations between cases detected postvaccination and among unvaccinated cases.

## METHODS

At BMC, HCWs are screened daily for COVID-19 symptoms and tested if symptomatic. Asymptomatic testing is available to HCWs for workplace exposures, following out-of-state travel, and per request. Routine asymptomatic serial screening for SARS-CoV-2 infection was not performed during this period. All HCWs diagnosed with SARS-CoV-2 complete contact tracing and clinical questionnaires. As BMC was a BNT162b2 trial site, 66 HCWs had been vaccinated prior to the vaccine initiative and were included in analyses. The BMC COVID-19 vaccine initiative ultimately included all HCWs; however, rollout was staged, with patient-facing and employees caring for SARS-CoV-2–positive patients offered vaccination first.

We identified all HCWs with SARS-CoV-2 by reverse-transcription polymerase chain reaction (RT-PCR) between 9 December 2020 and 23 February 2021. HCWs who received a vaccination following their positive SARS-CoV-2 RT-PCR were included in the unvaccinated group. We compared demographics and characteristics of cases detected postvaccination by time since first dose to RT-PCR positive (1–14 days and ≥15 days from first dose to RT-PCR) using Pearson χ ^2^, Fisher exact, and Student *t* tests. RT-PCR cycle threshold was dichotomized as above (negative) or below (positive) 24, the published cycle threshold above which SARS-CoV-2 has not been readily cultured, across 5 instruments (Supplementary Table 1) [5–7]. We computed the crude weekly case rates by vaccination status at the time of the tests (unvaccinated, 1–14 days, and ≥15 days from first dose to RT-PCR) as the number of weekly cases divided by person-days of follow-up during that week (see Supplementary Data for complete methodology). To control for confounding from community trends in infection, we adjusted the rates using direct standardization to the weekly rates in Middlesex, Norfolk, and Suffolk counties in Massachusetts [1]. We compared the weekly decline in rates after 30 December 2020 (14 days after the vaccine initiative started) for BMC HCWs and the community using a negative binomial regression model including an interaction term between week and group (BMC HCW vs community) and an offset for person-days at risk.

Residual isolates available from HCW SARS-CoV-2 cases tested at BMC were amplified using a modified ARTIC primer–based protocol and sequenced on an Illumina platform. Nucleotide substitutions, insertions, and deletions were identified with LoFreq [8] following alignment to the Wuhan-Hu-1 reference sequence (NC_045512.2) [9] with Bowtie2 [10]. We used a quality threshold of ≥10 reads for determining a change from reference, and low coverage sites were replaced with a placeholder in the consensus sequence. For the time-matched subanalysis, we then restricted to sequences from cases between 1 January and 23 February 2021. The date of the first case in the ≥15 days from first vaccine dose to RT-PCR group was 1 January 2021, so by excluding sequences from cases 1–14 days from first dose to RT-PCR and unvaccinated cases from before this date, we were able to control for the expected accumulation of mutations with time. Significance calculations for synonymous and nonsynonymous mutations between vaccination groups used a Wilcoxon nonparametric test. We further analyzed unique amino acid substitutions found in the spike protein of viruses isolated from cases ≥15 days after vaccination that were not found in unvaccinated cases, using initially an unmatched permutation analysis (Supplementary Figure 2*A*) and then a date-matched permutation analysis (Supplementary Figure 2*B*). Unrooted, maximum likelihood phylogenetic trees were inferred using IQ-TREE [11], and a GTR + F + I + G4 model of rate heterogeneity was chosen using the ModelFinder feature [12]. Tree manipulation and annotation were conducted in R software (R-project.org) [13, 14].

## RESULTS

**Figure 1.**
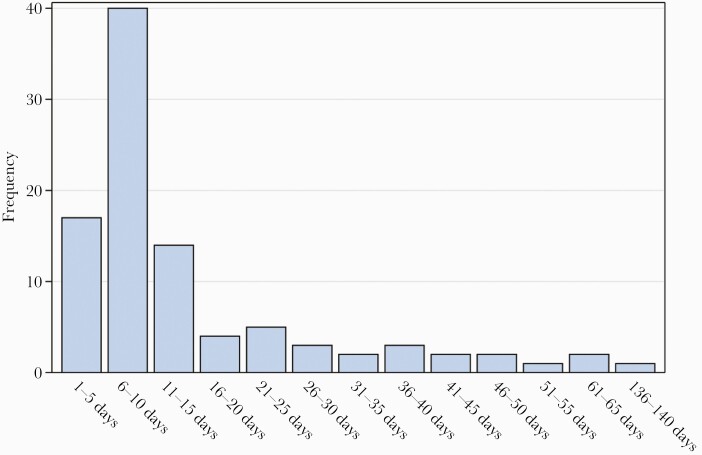
Time elapsed from first dose of severe acute respiratory syndrome coronavirus 2 vaccination to positive reverse-transcription polymerase chain reaction.

During the study period, 67% (7109/10 590) of eligible HCWs were vaccinated with at least 1 dose. Postvaccination SARS-CoV-2 cases occurred in 96 of 7109 (1.3%) HCWs who received at least 1 dose, 17 of 5913 (0.3%) HCWs given both doses, and 329 of 3481 (9.5%) unvaccinated HCWs. Seventy percent (67/96) of postvaccination SARS-CoV-2 cases occurred within 14 days of the initial dose (Figure 1). Comparison of HCW characteristics stratified by COVID-19 vaccination status at the time of SARS-CoV-2–positive RT-PCR are presented in Table 1, with post–initial vaccine dose cases more frequent among doctors and nurses compared to unvaccinated HCW cases (*P* < .01; Table 1). Among those who were vaccinated, HCWs diagnosed with SARS-CoV-2 ≥15 days after first dose were more often older (46 vs 38 years, *P* = .007), Latinx ethnicity (10% vs 8%, *P* = .03), asymptomatic (48% vs 11%, *P* = .0002), and afebrile (100% vs 84%, *P* = .02) and trended toward receipt of BNT162b2 (69% vs 48%, *P* = .06), compared to HCWs diagnosed within 14 days of first dose (Table 2). There was no difference in RT-PCR cycle threshold. The adjusted rate ratios (RRs) of SARS-CoV-2 infection were 0.73 (95% confidence interval [CI], .53–1.00) and 0.18 (95% CI, .10–.32) for 1–14 days and ≥15 days, respectively, from first dose compared to unvaccinated follow-up (Table 3).

**Table 1.** Employee Characteristics Stratified by Vaccination Status at Time of Positive Test

Characteristic	Vaccination Status at Time of Positive Test			*P* Value
	Total (N = 425)	Unvaccinated (n = 329)	Post–First Vaccine Dose (n = 96)	
Age, y, mean (SD) (n = 424)	40 (13)	39 (13)	40 (13)	.53
Role				<.01
Health care support worker	56 (14)	47 (15)	9 (10)	
Nurse	95 (24)	64 (21)	31 (33)	
Nurse practitioner/physician assistant	9 (2)	4 (1)	5 (5)	
Administrative staff	42 (10)	39 (13)	3 (3)	
Environmental services	7 (2)	5 (2)	2 (2)	
Physician	28 (7)	15 (5)	13 (14)	
Medical technician	22 (5)	16 (5)	6 (6)	
OT, PT, or speech therapist	2 (1)	1 (0.3)	1 (1)	
Pharmacy worker	19 (5)	14 (5)	5 (5)	
Respiratory therapist	4 (1)	2 (1)	2 (2)	
Other	120 (30)	103 (33)	17 (18)	
Test reason (n = 375)				.48^a^
Community exposure	130 (31)	102 (31)	28 (29)	
Hospital exposure	30 (7)	21 (6)	9 (9)	
Unknown exposure	215 (51)	159 (48)	56 (93)	
Asymptomatic (n = 375)	88 (21)	68 (21)	20 (21)	.67^a^
Fever	41 (10)	30 (9)	11 (12)	.49

Data are presented as No. (%) unless otherwise indicated.

Abbreviations: OT, occupational therapist; PT, physical therapist; SD, standard deviation.

^a^Fisher exact test, otherwise Pearson χ ^2^ test or Student *t* test as appropriate.

**Table 2.** Employee Characteristics Stratified by Vaccination-to-Diagnosis Timing

Characteristic	Total (N = 96)	Days Between First Dose of Vaccine and Positive Test		*P* Value^a^
		1–14 Days (n = 67)	≥15 Days (n = 29)	
Female	78 (82)	54 (82)	24 (83)	1.00^a^
Age, y, mean (SD)	40 (13)	38 (13)	46 (13)	.007
Ethnicity				
Latinx	8 (8)	5 (8)	3 (10)	.03^a^
Race				.12
Asian/Asian Indian	9 (10)	6 (9)	3 (10)	
Black/African American	21 (22)	15 (23)	6 (21)	
Hispanic or Latino	6 (6)	5 (8)	1 (3)	
Native Hawaiian/Pacific Islander	1 (1)	0 (0)	1 (3)	
White	50 (53)	37 (57)	13 (45)	
Unknown/declined	7 (7)	2 (3)	5 (17)	
Role				.16
Health care support worker	9 (10)	5 (8)	4 (14)	
Nurse	31 (33)	25 (38)	6 (21)	
Nurse practitioner/physician assistant	5 (5)	4 (6)	1 (4)	
Administrative staff	3 (3)	3 (5)	0 (0)	
Environmental services	2 (2)	2 (3)	0 (0)	
Physician	13 (14)	6 (9)	7 (25)	
Medical technician	6 (6)	5 (8)	1 (4)	
OT, PT, or speech therapist	1 (1)	1 (2)	0 (0)	
Pharmacy worker	5 (5)	3 (5)	2 (7)	
Respiratory therapist	2 (2)	0 (0)	2 (7)	
Other	17 (18)	12 (18)	5 (18)	
Type of vaccine				.06
BNT162b2	52 (54)	32 (48)	20 (69)	
mRNA-1273	44 (46)	35 (52)	9 (31)	
RT-PCR cycle threshold^b^				.99
>24	25 (26)	18 (27)	7 (24)	
≤24	39 (40)	28 (42)	11 (38)	
Missing	32 (33)	21 (31)	11 (38)	
Test reason (n = 93)				.79^a^
Community exposure	28 (30)	19 (29)	9 (34)	
Hospital exposure	9 (10)	6 (9)	3 (11)	
Unknown exposure	56 (60)	41 (62)	15 (56)	
Asymptomatic	20 (22)	7 (11)	13 (48)	.0002^a^
Fever	11 (12)	11 (16)	0 (0)	.02

Data are presented as No. (%) unless otherwise indicated.

Abbreviations: OT, occupational therapist; PT, physical therapist; RT-PCR, reverse-transcription polymerase chain reaction; SD, standard deviation.

^a^Fisher exact test, otherwise Pearson χ ^2^ or Student *t* test as appropriate.

^b^Twenty-four was the lowest cycle threshold above which severe acute respiratory syndrome coronavirus 2 was unable to be cultured [5–7].

**Table 3.** Severe Acute Respiratory Syndrome Coronavirus 2 Rate Reductions Among Boston Medical Center Health Care Workers by Vaccination Status^a^

SARS-CoV-2 Cases	Unvaccinated^a^	Days Between First Dose of Vaccine and Positive Test	
		1–14 Days	≥15 Days
No. of cases	329	67	29
Person-days at risk	406 387	96 041	251 790
Crude rate per 100 000 person-days at risk (95% CI)	80.96 (72.44–90.20)	69.76 (54.06–88.60)	11.52 (7.71–16.54)
Adjusted rate per 100 000 person-days at risk (95% CI)	71.64 (63.22–80.07)	50.02 (35.47–64.57)	12.69 (5.29–20.09)
Adjusted rate ratio (95% CI)	Ref	0.73 (.53–1.00)	0.18 (.10–.32)

Abbreviations: CI, confidence interval; SARS-CoV-2, severe acute respiratory syndrome coronavirus 2.

^a^See Supplementary Data for full methodology.

Following COVID-19 vaccine rollout to HCWs, case rates fell rapidly to below those of the community, to whom vaccine was largely not yet available (Supplementary Figure 1). Though not statistically significant, the observed weekly decrease in case rates was faster among BMC HCWs than in the community (HCW RR, 0.82; community RR, 0.89; *P* = .14).

**Figure 2.**
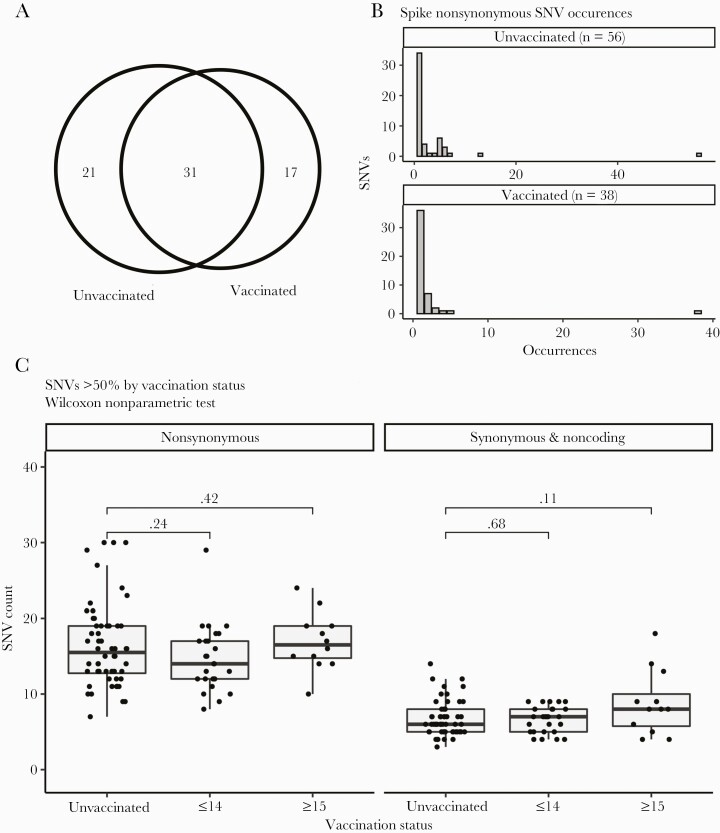
Severe acute respiratory syndrome coronavirus 2 (SARS-CoV-2) genome sequence variation among time-matched vaccinated and unvaccinated positive SARS-CoV-2 reverse-transcription polymerase chain reaction (RT-PCR) health care worker (HCW) cases. *A*, Venn diagram illustrating how the nonsynonymous single-nucleotide variants (SNVs) in spike are distributed. A total of 69 SNVs were identified in 94 time-matched sequenced genomes. Forty-eight different SNVs were distributed over 38 genomes from individuals with a positive SARS-CoV-2 RT-PCR following at least their first vaccination. Fifty-two SNVs were distributed among 56 genomes from unvaccinated cases. Thirty-one SNVs were identified in genomes from both vaccinated and unvaccinated individuals. *B*, Distribution of spike SNVs between genomes in vaccinated and unvaccinated populations. *C*, Box-and-whisker plots illustrating the number of nonsynonymous mutations found in infected HCWs who were unvaccinated, infected within 14 days after initial vaccine dose (≤14), or infected ≥15 days after initial vaccine dose as compared to the Wuhan-Hu-1 sequence. Significance calculations were calculated using Wilcoxon nonparametric test.

We were able to successfully sequence isolates from 52% (50/96) of SARS-CoV-2 cases diagnosed after at least the first vaccine dose. Based on the inclusion criteria described above, sequences from 38 post–first vaccine SARS-CoV-2 cases and 56 unvaccinated cases were included in the time-matched subanalysis. Whole genome analysis identified 69 total nonsynonymous single-nucleotide variants (SNVs) in the spike protein, with 52 and 48 SNVs among unvaccinated and after at least initial vaccine isolates (Figure 2A). Of these SNVs, 31 were seen in both the unvaccinated and vaccinated cases, consistent with these infections occurring in an environment where similar variants were circulating. To determine whether there was greater accumulation of SNVs in SARS-CoV-2 spike gene from either unvaccinated or vaccinated groups, we plotted the distribution of nonsynonymous SNVs between genomes in each population (Figure 2B). This analysis showed that most SNVs were found in a single genome from an individual case, with little accumulation of multiple mutations in either population. The exception to this trend was 3 mutations associated with the D614G variant that appeared in all genomes analyzed.

Analysis of the amino acid changes seen in vaccinated cases identified no selection for the antibody-evading E484K mutation. There were 2 amino acid changes (T1117A and N121D) seen in 2 separate postvaccination infections and not seen in unvaccinated cases (Supplementary Table 2). Multiple separate acquisitions of the same mutation would suggest convergent evolution; however, phylogenetic analysis showed that in both cases these were 2 separate infections with the same closely related lineage (Supplementary Figure 3). While genome sequences from cases among vaccinees ≥15 days post–first dose appeared more divergent from the original Wuhan-Hu-1 reference when compared with unvaccinated, this was no longer the case after controlling for the date of sampling; more recent samples are more divergent because more time has elapsed in which to accumulate mutations (Figure 2C). We further identified unique amino acid substitutions found in the spike protein of viruses isolated from cases ≥15 days after vaccination that were not found in unvaccinated cases (Supplementary Table 2). Although slightly fewer (*P* > .05) spike substitutions were observed in viruses isolated from cases ≥15 days after initial vaccine dose, adjusted for sampling date (Figure 2C), we hypothesized that vaccine-induced selection pressure could be driving novel mutations. While an initial unmatched permutation analysis showed a significant difference (Supplementary Figure 2*A*), a second date-matched permutation analysis was conducted and we concluded that the number of unique spike substitutions in viruses isolated from cases ≥15 days after the first dose was not significantly different than what would be expected by chance (Supplementary Figure 2*B*).

## DISCUSSION

Our findings suggest that SARS-CoV-2 vaccines decreased case rates at a time of high community prevalence and domestic circulation of concerning variants [15]. While postvaccination SARS-CoV-2 cases do occur, the majority are in the 2 weeks following the first dose. We found adjusted rate reduction of new cases in vaccinated HCWs in days 1–14 and ≥15 days post–first dose vs unvaccinated of 27% and 82%, respectively, similar to findings in the original clinical trials and more recently reported in Israel, the United Kingdom, and other parts of the United States [16–22]. Vaccine protection appears to start to have a greater impact 2 weeks after the first vaccination dose. Individuals infected >2 weeks after first vaccination dose compared to before 2 weeks from first dose were older and reported fewer symptoms.

While we observed a decrease in rates among vaccinated vs unvaccinated individual HCWs, we did not observe a significant decrease in rates among HCWs vs the community in the months following the HCW vaccination initiative. Community rates likely surged in December due to holiday gatherings and travel, then trended down as individuals returned to more normal social distancing after the holiday season. Such behavior changes may have led to some of the HCW case rate drop as well, but the decline was steeper among HCWs, likely reflecting vaccine impact. The HCW infection rates then flattened to falling community rates, even at a time when few members of the community were eligible for vaccine. HCWs at greatest risk for contracting SARS-CoV-2 infection outside of the workplace may have chosen not to receive vaccine, something we could not adjust for with our data, but could lead to the plateau in population-level effectiveness. Similarly, that we saw more postvaccination cases among doctors and nurses likely reflects higher uptake of vaccine among HCWs with those roles. We were unable to adjust for markers such as a social vulnerability index; however, disparities in vaccination uptake exist [23] and may lead to reduced vaccine effectiveness when compared with expectations from trial results. Even when access is not an obstacle, as in the case of HCWs, vaccine hesitancy and other barriers will need to be better understood and addressed to reach the levels of population immunity necessary to slow the spread of SARS-CoV-2 infection. In the meantime, mitigation measures such as masking, physical distancing, ventilation, and regular testing are still needed in hospital settings to protect both staff and patients. This is supported by recent modeling data suggesting that to avoid future outbreaks some mitigation measures will need to be maintained in the community at large even when high vaccine coverage is attained, as a result of the circulation of more transmissible variants [24].

Our data lacked follow-up from initial SARS-CoV-2 diagnosis, so initially asymptomatic cases may have progressed to symptomatic disease in all groups. Additionally, though HCWs diagnosed with SARS-CoV-2 ≥15 days from their initial dose were more likely to have received the BNT162b2 vaccine, this result likely reflects that BNT162b2 was given early when the force of infection was highest. Without routine asymptomatic screening for SARS-CoV-2 among BMC HCWs during this time period, it is possible that unvaccinated BMC HCWs were more likely to seek out asymptomatic testing than those who had received vaccine, leading to identification of more unvaccinated cases. Additional limitations of the study are that a larger sample size is likely needed to assess the statistical significance of the adjusted RRs of SARS-CoV-2 infection at 1–14 days and deferral bias, as seen in prior studies, [22] may have contributed to a lower rate ratio in this early group. Larger datasets should be used to compare the potential confounding, such as the impact of age on symptom severity, among cases following vaccination, which our small sample size prohibited. Finally, we are limited by our ability to evaluate only for vaccine-related mutations in the consensus sequence; future studies could use further spike gene amplification to allow for detection of low-frequency variants.

There is intense interest in, and anxiety over, the potential of vaccination to select for mutants against which vaccines are less effective [25]. We found no early evidence of selection for specific spike mutations or mutations associated with neutralizing vaccine escape, such as the E484K mutation. We observed overall genome divergence relative to the Wuhan-Hu-1 sequence as expected, with no evident increase of greater divergence in the spike protein. Our study compared cases among vaccinees with those of contemporaneous unvaccinated individuals in the same population, which reflect the pool of virus to which vaccinated individuals were exposed, in order to avoid spurious associations between SNVs that happen to be locally common in different populations. It is a strength of this study that such time-matched samples were available. Future studies should aim for similar design, as we had dramatically different findings when the unique spike protein substitution permutation analysis was conducted without date matching (Supplementary Figure 2). We were also able to stratify participant samples by weeks from initial vaccine dose, when significant anti-spike antibody has been demonstrated to have developed [16, 26]. Not simply stratifying dichotomous vaccinated or unvaccinated samples allowed observation of whether building immunity impacts the virus. Although we did not see clear trends, variation in the spike sequence among vaccinated cases should be closely monitored by studies with larger sample size, with careful consideration given to vaccine timing and comparison to concurrent unvaccinated controls.

Widespread vaccination does appear to impact case rates in real-world campaigns. We must continue to improve vaccine coverage for all globally, educate about the need for vigilance in social distancing in the weeks following first dose, and carefully monitor for any ongoing viral evolution that impacts vaccine efficacy.

## Supplementary Data

Supplementary materials are available at *Open Forum Infectious Diseases* online. Consisting of data provided by the authors to benefit the reader, the posted materials are not copyedited and are the sole responsibility of the authors, so questions or comments should be addressed to the corresponding author.

ofab465_suppl_Supplementary_Figures

ofab465_suppl_Supplementary_Materials

## Notes

***Acknowledgments.*** The authors thank John Goldie for helping with the Boston Medical Center (BMC) vaccination data; BMC for providing financial support for this study; and all the patients and staff involved in the BMC COVID-19 efforts this year. The biologic samples used for the analyses presented here were obtained from the BMC/Boston University COVID-19 Biorepository.

***Patient consent statement.*** Boston University Medical Campus’ institutional review board reviewed the study and found it qualified for an exemption determination.

***Financial support.*** This work was supported by the National Institutes of Health (service award number T32-DA013911 to T.C.B. cooperative agreement number 1UL1TR001430 to K. R. J.); and the Burroughs Wellcome Fund/American Society for Tropical Medicine and Hygiene Postdoctoral Fellowship in Tropical Infectious Diseases (to T. C. B.).

***Potential conflicts of interest.*** All authors: No reported conflicts of interest.
