# Supplementary figures and images for: Coronavirus Disease 2019 Vaccine Impact on Rates of Severe Acute Respiratory Syndrome Coronavirus 2 Cases and Postvaccination Strain Sequences Among Health Care Workers at an Urban Academic Medical Center: A Prospective Cohort Study
Source: Open Forum Infect Dis. 2021 Sep 17;8(10):ofab465. doi: 10.1093/ofid/ofab465 (PMC8500299; doi:10.1093/ofid/ofab465)

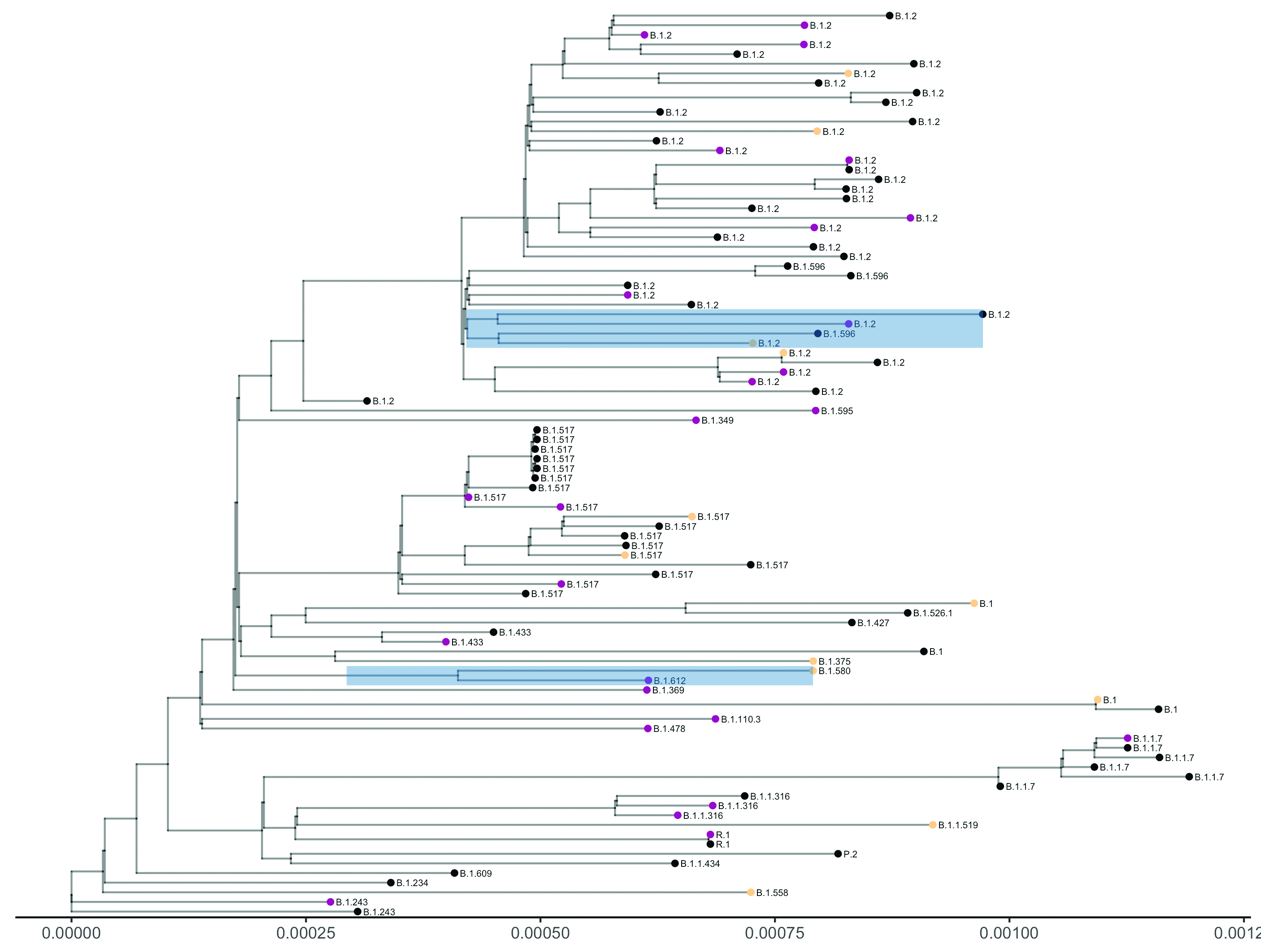

Supplement: ofab465_suppl_Supplementary_Figures [file ofab465_suppl_supplementary_figures.zip › ofab465_suppl_Supplementary_Figure_S3.tif]

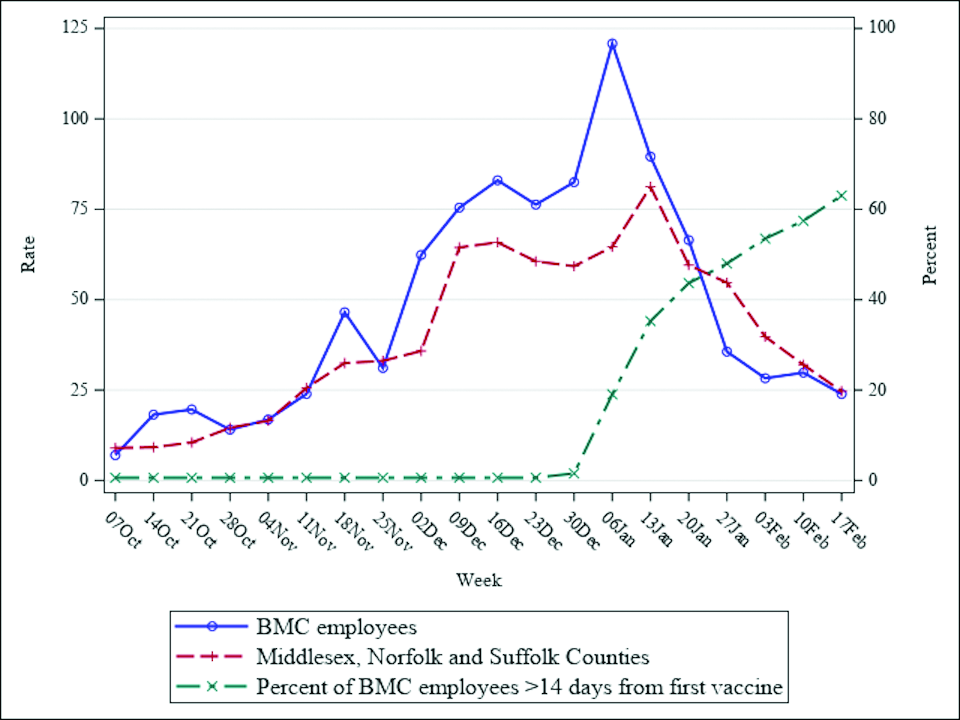

Supplement: ofab465_suppl_Supplementary_Figures [file ofab465_suppl_supplementary_figures.zip › ofab465_suppl_Supplementary_Figure_S1.tiff]
