# Supplementary material for: Coronavirus Disease 2019 Vaccine Impact on Rates of Severe Acute Respiratory Syndrome Coronavirus 2 Cases and Postvaccination Strain Sequences Among Health Care Workers at an Urban Academic Medical Center: A Prospective Cohort Study
Source: Open Forum Infect Dis. 2021 Sep 17;8(10):ofab465. doi: 10.1093/ofid/ofab465 (PMC8500299; doi:10.1093/ofid/ofab465)
Supplement: ofab465_suppl_Supplementary_Materials [file ofab465_suppl_supplementary_materials.docx]

**Supplementary Data**

SARS-CoV-2 Rate Reductions Among BMC HCW by Vaccination Status: Full Methodology

Number of cases while unvaccinated, 1-14 days post vaccination and >14 days post vaccination were specified by week between December 9^th^, 2020 and February 24^th^, 2021. For each BMC employee, person-day of follow-up at risk while unvaccinated started on December 9^th^, 2020 and ended at the earliest of infection date, date of first vaccination shot, or February 24^th^, 2021. Person-day follow-up at risk 1-14 days post vaccination started on the day after the first vaccination shot and ended at the earliest of infection date, the date corresponding to 14 after the first vaccination shot or end of the study. Person-day follow-up at risk >14 days post vaccination started 15 days after the first vaccination shot and ended at the earlier of infection date, or end of the study. Infection rates by vaccination status (unvaccinated, 1-14 post vaccination and >14 days post-vaccination) were expressed as number of cases per 100,000 person-day follow-up at risk overall and by week. Adjustment for temporal trends of infection in the community was performed by direct standardization to the weekly rates in 3 counties including and surrounding Boston where most BMC employees live. Directly standardized rates of the study population (unvaccinated BMC employees) are weighted averages of the weekly rates, where the weights are corresponding strata population size in the community. Weekly cases and estimated population in the 3 counties were obtained from the Massachusetts Department of Public Health publicly available dashboard and US Census Bureau, respectively and were used to compute the weekly infection rates in the community as number of cases per 100,000 person-days at risk.

Supplementary Figure 1: Weekly Rates of SARS-CoV-2 Cases per 100,000 Individuals and percent of vaccinated healthcare workers

Supplementary Table 1: SARS-CoV-2 Platforms Used To Diagnose via RT-PCR, Cycle Threshold Values^a^

| Platform, No. (%) | Total (N=64) | Days Between First Dose of Vaccination and Positive Test | |
| --- | --- | --- | --- |
|  |  | **1-14 days (N=46)** | **15+ days**  **(N=18)** |
| Abbott m2000 (SARS-CoV-2) | 4 (6) | 3 (7) | 1 (6) |
| Roche cobas 6800 (cobas SARS-CoV-2) | 2 (3) | 2 (4) | 0 (0) |
| Genetic Signatures GS1-HT (EasyScreen SARS-CoV-2) | 45 (70) | 29 (63) | 16 (89) |
| DiaSorin Simplexa (SARS-CoV-2) | 9 (14) | 8 (17) | 1 (6) |
| Cepheid GeneXpert (SARS-CoV/Flu/RSV) | 4 (6) | 4 (9) | 0 (0) |

^a^Data includes all cases post-vaccination for which a specimen was identified, and cycle threshold value successfully obtained from diagnostic platform.

Supplementary Table 2. Nonsynonymous mutations found only in the Spike protein of isolates from post vaccination cases (N=50)

| **Amino Acid Mutations** | **Count** |
| --- | --- |
| N121D | 2 |
| T1117A | 2 |
| L8V | 1 |
| L18F | 1 |
| W152L | 1 |
| Q173K | 1 |
| Q271E | 1 |
| E309Q | 1 |
| P322S | 1 |
| T478K | 1 |
| N616D | 1 |
| A701V | 1 |
| T732A | 1 |
| E780Q | 1 |
| A845V | 1 |
| T859I | 1 |
| K1073R | 1 |
| H1101Y | 1 |
| I1232V | 1 |

Supplementary Figure 2. Permutation analysis of unique amino acid substitutions found in the spike protein of viruses isolated from cases >14 days after first vaccine dose

A) Unmatched permutation analysis of unique spike amino acid (AA) substitutions found in the spike protein of viruses isolated from cases diagnosed ≥15 days from first vaccine dose that were not found in unvaccinated cases. Red vertical line indicates observed value of **11** unique spike AA substitutions (p < 0.001). B) Date-matched permutation analysis of unique spike amino acid (AA) substitutions found in the spike protein of viruses isolated from cases diagnosed ≥15 days from first vaccine dose that were not found in unvaccinated cases. Red vertical line indicates observed value of **11** unique spike AA substitutions (p = 0.1625). Data is from time-matched analysis.

Supplementary Figure 3. Phylogenetic tree of sequenced cases

Phylogenetic tree of sequenced cases with tip colors indicating case vaccination status and labels denoting viral PANGO[27] lineage designations. Clades highlighted in blue indicate location of T1117A (two-member clade) and N121D (four-member clade) substitutions.

Black tips = unvaccinated

Purple tips = ≤ 14 days from vaccination

Yellow tips = ≥ 15 days from vaccination

Tip labels = PANGO lineage designations
